# Metabolic Disorders and Male Hypogonadotropic Hypogonadism

**DOI:** 10.3389/fendo.2019.00345

**Published:** 2019-07-25

**Authors:** Rosario Pivonello, Davide Menafra, Enrico Riccio, Francesco Garifalos, Marco Mazzella, Cristina de Angelis, Annamaria Colao

**Affiliations:** Dipartimento di Medicina Clinica e Chirurgia, Sezione di Endocrinologia, Centro di Andrologia, Medicina della Riproduzione e della Sessualità Maschile e Femminile (FERTISEXCARES), Università “Federico II” di Napoli, Naples, Italy

**Keywords:** hypogonadotropic hypogonadism, metabolic disorders, type-2 diabetes mellitus, obesity, metabolic syndrome, testosterone, body composition, insulin resistance

## Abstract

Several studies highlight that testosterone deficiency is associated with, and predicts, an increased risk of developing metabolic disorders, and, on the other hand, is highly prevalent in obesity, metabolic syndrome and type-2 diabetes mellitus. Models of gonadotropin releasing hormone deficiency, and androgen deprivation therapy in patients with prostate cancer, suggest that hypogonadotropic hypogonadism might contribute to the onset or worsening of metabolic conditions, by increasing visceral adiposity and insulin resistance. Nevertheless, in functional hypogonadism, as well as in late onset hypogonadism, the relationship between hypogonadotropic hypogonadism and metabolic disorders is bidirectional, and a vicious circle between the two components has been documented. The mechanisms underlying the crosstalk between testosterone deficiency and metabolic disorders include increased visceral adipose tissue and insulin resistance, leading to development of metabolic disorders, which in turn contribute to a further reduction of testosterone levels. The decrease in testosterone levels might be determined by insulin resistance-mediated and, possibly, pro-inflammatory cytokine-mediated decrease of sex hormone binding globulin, resulting in a temporary increased free testosterone available for aromatization to estradiol in visceral adipose tissue, followed by a subsequent decrease in free testosterone levels, due to the excess of visceral adipose tissue and aromatization; by a direct inhibitory effect of increased leptin levels on Leydig cells; and by a reduced gonadotropin secretion induced by estradiol, inflammatory mediators, leptin resistance, and insulin resistance, with the ultimate determination of a substantial hypogonadotropic hypogonadism. The majority of studies focusing on the effects of testosterone replacement therapy on metabolic profile reported a beneficial effect of testosterone on body weight, waist circumference, body mass index, body composition, cholesterol levels, and glycemic control. Consistently, several interventional studies demonstrated that correction of metabolic disorders, in particular with compounds displaying a greater impact on body weight and insulin resistance, improved testosterone levels. The aim of the current review is to provide a comprehensive overview on the relationship between hypogonadotropic hypogonadism and metabolism, by clarifying the independent role of testosterone deficiency in the pathogenesis of metabolic disorders, and by describing the relative role of testosterone deficiency and metabolic impairment, in the context of the bidirectional relationship between hypogonadism and metabolic diseases documented in functional hypogonadotropic hypogonadism. These aspects will be assessed by describing metabolic profile in men with hypogonadotropic hypogonadism, and androgenic status in men with metabolic disorders; afterwards, the reciprocal effects of testosterone replacement therapy and corrective interventions on metabolic derangements will be reported.

## Introduction

Hypogonadism is a clinical condition characterized by an impairment of gonadal function; in men, the condition implies the presence of testosterone deficiency, with circulating testosterone levels below the normal range, often accompanied by an impairment of spermatogenesis, and the presence of a clinical syndrome mainly characterized by sexual dysfunction, generalized asthenia, reduction of testis volume, and gynecomastia, together with anemia and deterioration of muscle mass and strength and bone status (1). Moreover, hypogonadism is often associated with metabolic comorbidities, such as obesity, insulin resistance (IR), metabolic syndrome (MetS), and type-2 diabetes mellitus (T2-DM) (2).

Hypogonadism is classified as primary, if the underlying cause of the endocrine dysfunction is testicular failure, or central, if the underlying cause of the endocrine disorder relies on the hypothalamus-pituitary dysfunction. In primary hypogonadism, gonadotropins levels are generally increased, and the condition is defined hypergonadotropic hypogonadism (Hyper-H), whereas in central hypogonadism, gonadotropins levels are decreased, and the condition is defined hypogonadotropic hypogonadism (Hypo-H) (1). Hypothalamic and pituitary non-functioning and functioning tumors, hyperprolactinemia, pituitary surgery and irradiation, cranial trauma causing stalk injury, infiltrative diseases, such as hemochromatosis, sarcoidosis, and histiocytosis X, infectious pituitary lesions, acute systemic illness, medications including gonadotropin releasing hormone (GnRH) agonists or antagonists, opioids, and glucocorticoids, eating disorders, excessive exercise, and abuse of alcohol or addictive drugs may determine an acquired form of Hypo-H (1). Conversely, congenital Hypo-H is a rare pathological condition associated with a congenital deficiency of hypothalamic GnRH release, which secondarily induce testosterone deficiency (3). Lastly, a different form of hypogonadism is represented by late onset hypogonadism (LOH), a pathological condition occurring in middle-aged and elderly men, characterized by testosterone deficiency and low or normal gonadotropins levels, and a specific clinical syndrome, which is often associated with, or caused, by age-related metabolic disorders (4). In the last few years, a different classification of hypogonadism has emerged, based on the concept of “organic hypogonadism,” as opposed to the concept of “functional hypogonadism” (5–7), the latter being defined as “a condition with no recognizable structural intrinsic hypothalamus-pituitary-testis axis pathology and no specific pathologic conditions suppressing the axis” (5–7). In functional hypogonadism, testosterone levels are borderline and fluctuate around the lower limit of normality, and might be occasionally severely low, whereas gonadotropins are usually within the normal range, occasionally below the normal range (6). A causal relationship between testosterone deficiency and the clinical syndrome of functional hypogonadism is uncertain, since it is more often associated to morbidities affecting the hypothalamus-pituitary-testis activity, such as obesity, MetS or T2-DM, or to aging, although the mild testosterone decline occurring with aging is considered mostly due to accumulation of morbidities (5–7).

A crucial aspect of Hypo-H is represented by a strict link between testosterone deficiency and metabolic disorders, which, especially in LOH and functional hypogonadism, is characterized by a bidirectional relationship. Indeed, observational studies and metanalyses demonstrated that hypogonadism, in particular testosterone deficiency, is associated with metabolic disorders, and predicts an increased risk of developing incident MetS (8–11) and T2-DM (11–13). On the other hand, observational studies demonstrated that obesity, mainly characterized by visceral adiposity, IR (14, 15), MetS (14–16), as well as T2-DM (14, 17, 18) are often associated with testosterone deficiency and predicts an increased risk of developing incident hypogonadism.

Moreover, additional studies highlighted that the association of testosterone deficiency with metabolic disorders frequently occurs in a clinical context of Hypo-H, and that Hypo-H is frequently reported in metabolic disorders, such as obesity, IR, MetS, and T2-DM (15, 19, 20). LOH is typically associated with metabolic disorders (4, 21), and has been hypothesized to link obesity, IR and MetS, and T2-DM to sexual dysfunction, the most common symptom of LOH (2, 22). Moreover, in LOH, a bidirectional relationship has been described between hypogonadism, which contributes to increase visceral obesity, and obesity, which contributes to worsen the condition of Hypo-H (23).

Treatment options for Hypo-H and its related metabolic disorders include testosterone replacement therapy (TRT), as well as alternative therapeutic approaches, suitable to contemporarily normalize testosterone levels and restore fertility. These alternative options include human chorionic gonadotropin, and off-label therapies, such as aromatase inhibitors and selective estrogen receptor modulators (24). Moreover, the insulin sensitizer metformin has been reported to improve not only metabolic profile but also androgenic status and semen quality in small interventional studies in men with impairment of insulin sensitivity (25) and in men with Hypo-H and impairment of spermatogenesis (26). These alternative therapies grant an increase of testosterone levels, combined to an improvement of semen quality, and, eventually, of metabolic profile, by avoiding the main side effects potentially linked to long-term TRT, which mainly include inhibition of spermatogenesis, pathological increase of erythropoiesis, and prostate hypertrophy (24). In particular, despite TRT was associated with a significant short-term increase in prostate volume, particularly in patients with severe pre-treatment testosterone deficiency, without risk of developing prostate cancer in long-term studies (5), given the androgen-responsive nature of prostate cancer, caution is recommended in the administration of TRT, in cases with overt or suspected prostate cancer, by limiting TRT only to symptomatic hypogonadal patients successfully treated for prostate cancer, after a prudent interval (27).

The current review aims at exploring the association between Hypo-H and metabolic disorders, by describing the potential, independent, pathogenetic roles of testosterone deficiency and/or metabolic alterations, in the context of the strict relationship between hypogonadism and metabolic diseases, which, in functional hypogonadism and LOH, has been described as a vicious circle. In summary, the current review describes: (1) the consequence of hypogonadism, and, particularly, testosterone deficiency, on metabolic profile; (2) the consequence of metabolic disorders on androgenic status; (3) the effects of TRT on metabolic profile; (4) the effects of metabolic disorders correction on androgenic status.

## Metabolic Disorders and Hypogonadotropic Hypogonadism: Observational Studies

Compelling evidences from longitudinal studies and metanalyses demonstrated that hypogonadism, in particular testosterone deficiency, is associated with metabolic disorders, and predicts an increased risk of developing MetS and T2-DM (8–13). A population-based prospective cohort study on 702 middle-aged men without MetS or T2-DM at baseline demonstrated that men with total testosterone (TT) or calculated free testosterone (FT) or sex hormone binding globulin (SHBG) levels in the lowest quartile had an increased risk of developing MetS, when compared with men in the higher quartiles, after 11 years of follow-up, although, after adjusting for potential confounders, including correlates of IR, such as body mass index (BMI), waist circumference (WC), insulin levels, and including components of MetS, such as glucose and triglyceride levels and systolic blood pressure, the association of calculated FT with increased risk for MetS was lost (11). A different study with a 15-years follow-up on 950 healthy aging men confirmed these results, by highlighting a progressive increase of odds for MetS along TT, FT, and SHBG levels quartiles, from the first to the fourth quartile (9). Likewise, an increased risk of developing T2-DM was demonstrated in healthy men in the lowest quartile compared to those in the highest three quartiles of TT levels (13).

The exact pathophysiological mechanism by which testosterone deficiency leads to metabolic impairment, therefore contributing to obesity, IR, MetS, and T2DM is still unclear. However, testosterone deficiency has been proposed to induce an increase in lipoprotein lipase activity, resulting in increased fatty acids uptake and triglyceride formation in adipocytes, which ultimately stimulate adipocyte proliferation and accumulation of adipose tissue, particularly visceral adipose tissue (VAT) (28, 29), therefore explaining the development of obesity, particularly visceral obesity, IR and MetS in patients with hypogonadism. Moreover, the upregulation of multiple mitochondrial enzymes, consequently determining a global impairment of mitochondrial function, has been proposed to contribute to the development not only of fatigue, but also IR, MetS, and T2-DM, in men with hypogonadism (30). On the other side, experimental animal models demonstrated that insulin exerts a stimulatory action on hypothalamic GnRH neurons, which results in gonadotropin release, therefore suggesting that IR associated with metabolic disorders might reduce gonadotropin secretion and promote hypogonadism (31). Moreover, VAT accumulation characterizing visceral obesity, results in increased aromatization of testosterone to estradiol (28, 30, 32) and enhanced action of inflammatory mediators, including tumor necrosis factor-a (TNF-a), interleukin-6 (IL-6), and interleukin-1b (IL-1b), shown to suppress hypothalamic GnRH, and, consequently, pituitary luteinizing hormone (LH) secretion in experimental *in vitro* and *in vivo* studies (33, 34), with induction of testosterone deficiency. Lastly, a decrease of testosterone levels is also promoted by leptin through a direct inhibitory effect on Leydig cells, as suggested by human models (35), as well as indirectly through a leptin-resistance mechanism at the hypothalamic-pituitary level, probably mediated by down-regulation of leptin receptor, as suggested by murine models (36).

A crucial role in the crosstalk between metabolic disorders and testosterone deficiency has been attributed to SHBG levels, which have been shown to be reduced in obese men (37) and men with T2-DM (38), as well as to be negatively associated with the risk of MetS and T2-DM (39). Moreover, visceral adiposity has been pointed out by a recent, large, prospective study, as being negatively correlated with SHBG levels (40). A crucial role for IR, and compensatory hyperinsulinemia, in the suppression of SHBG levels has been strongly supported by a clear inverse relationship between serum insulin and SHBG levels (38, 41, 42). Lastly, evidences from experimental and clinical studies demonstrated that SHBG is downregulated by pro-inflammatory cytokines, such as TNF-a and IL-1b (39), therefore suggesting that these factors might play a role in the reduction of SHBG levels in chronic inflammatory diseases, such as obesity and diabetes, characterized by increased levels of pro-inflammatory cytokines. The reduction of SHBG levels might result in temporary increase of FT levels, which might enhance aromatase activity, already increased by the VAT accumulation, therefore emphasizing the conversion to estradiol (41), which exerts a negative feedback on the HPT axis, ultimately inducing a reduction of TT and FT levels that remain associated with the reduction of SHBG levels.

**Figure 1:**
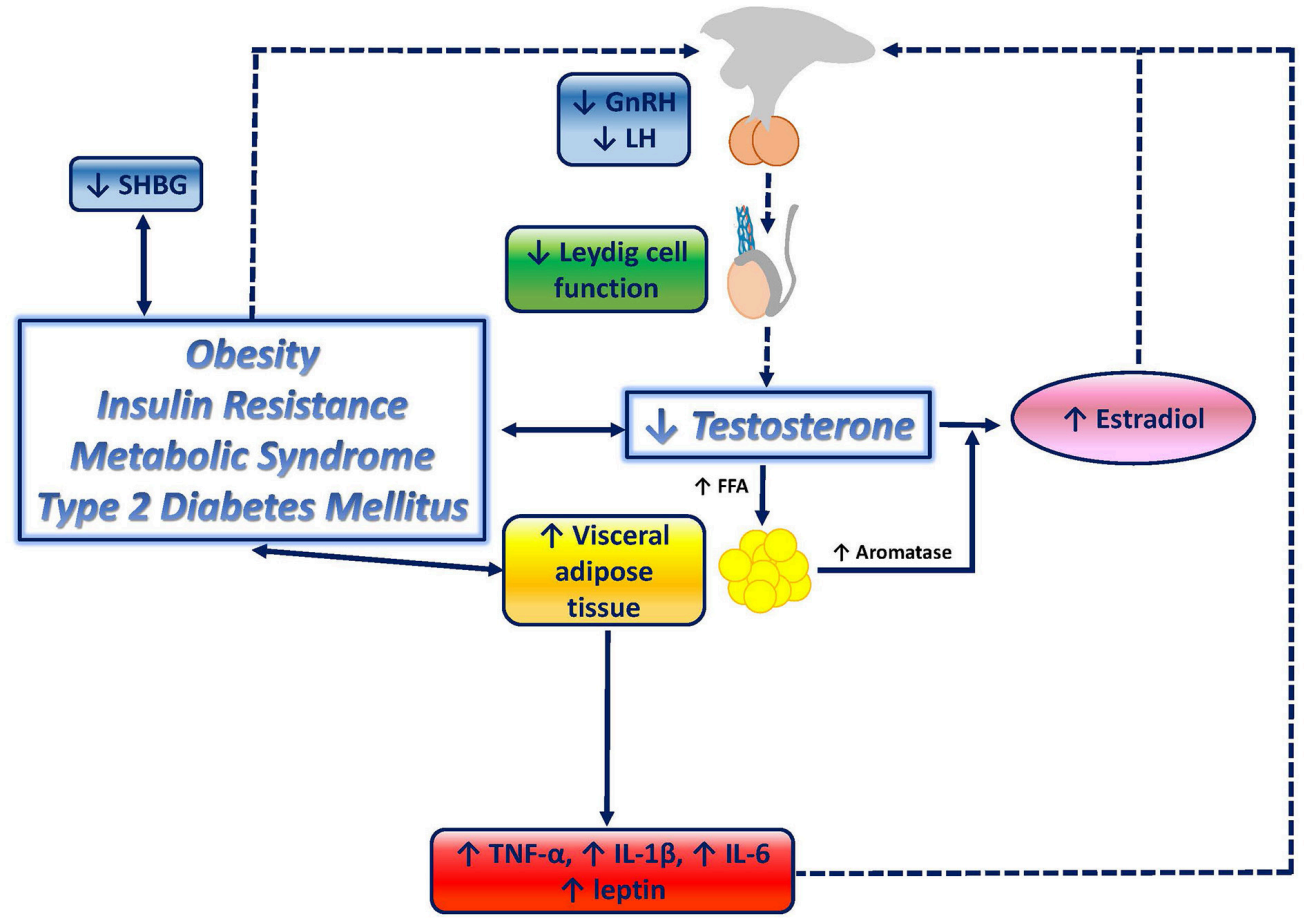
Graphical overview of the relationship between testosterone deficiency and metabolic disorders. Testosterone deficiency determines an increase in lipoprotein lipase activity, resulting in increased fatty acids (FFA) uptake and triglyceride levels in adipocytes, which ultimately stimulate adipocyte proliferation and visceral adipose tissue (VAT) increase, and therefore, contribute to the onset of visceral obesity. VAT accumulation triggers several downstream mechanisms which contribute to further impair the metabolic profile, and, potentially, enhances testosterone deficiency by closing the loop: increased aromatase activity results in increased conversion of testosterone to estradiol; inflammatory mediators, known to be increased in metabolic disorders, including tumor necrosis factor-a (TNF-a), interleukin-6 (IL-6), and interleukin-1b (IL-1b), have been shown to suppress hypothalamic gonadotropin releasing hormone (GnRH), and consequently, pituitary luteinizing hormone (LH) and testosterone secretion; increased leptin levels exert direct inhibitory effects on Leydig cells; leptin resistance at the hypothalamic-pituitary level, mediated by reduced LH release, boosts testosterone suppression; insulin resistance (IR) associated with obesity, metabolic syndrome and type-2 diabetes mellitus has been suggested to reduce gonadotropin secretion, therefore contributing to testosterone deficiency, and to reduce sex hormone binding globulin (SHBG).

In conclusion, testosterone deficiency promotes obesity, especially visceral obesity, IR, MetS and T2-DM, which in turn contribute to a further reduction of testosterone levels, determined by (1) IR-mediated and pro-inflammatory cytokine-mediated decrease of SHBG levels, ultimately resulting in negative feedback on the HPT axis; (2) direct inhibitory effect of increased leptin levels on Leydig cells; and (3) indirect inhibition due to HPT axis suppression induced not only by estradiol excess but also by inflammatory mediators, leptin resistance and IR. The combinations of these mechanisms induces the determination of a substantial Hypo-H. A graphical overview of the relationship between testosterone deficiency and metabolic disorders is depicted in Figure 1.

In the context of Hypo-H, few studies exist in organic, genetic or idiopathic forms of Hypo-H, focused on metabolic profile and on the potential independent role of testosterone deficiency in the development of metabolic disorders; nevertheless, hypogonadism due to deficient production, secretion, or action of GnRH, as well as pharmacologically-induced hypogonadism for androgen deprivation therapy (ADT), might represent suitable clinical models of Hypo-H, with available evidence of testosterone deficiency-induced metabolic disorders. Interestingly, one prospective cohort study on 12 young men with idiopathic GnRH deficiency demonstrated that insulin sensitivity was significantly reduced, and fasting insulin and IR, as assessed by HOMA-IR, were significantly increased, after 2 weeks of acute TRT withdrawal (43), demonstrating that testosterone deficiency might independently increase IR. Indeed, acute castration in patients with GnRH deficiency, achieved by withdrawing TRT, enables the assessment of the independent effects of acute changes in circulating testosterone levels, by dissecting the potentially confounding effects of changes in body composition and metabolism, which can be induced by long-term castration interventions (44), such as ADT with GnRH analogs (GnRHa), in men with androgen-sensitive prostate cancer (45–47). ADT-induced Hypo-H in men with prostate cancer has been associated with increased fat mass (45–47), decreased insulin sensitivity (45, 48), and increased risk of MetS and T2-DM (49). Moreover, differently from classical MetS, ADT-induced Hypo-H is associated with increased, rather than decreased, high-density lipoprotein (HDL) levels, and a preferential increase in subcutaneous adipose tissue, rather than VAT (47). Lastly, a large population-based cohort study on elderly men with prostate cancer demonstrated that ADT-induced Hypo-H was associated with a significantly increased risk of T2-DM (50). Further evidences supporting the hypothesis of a direct and independent role of testosterone deficiency in the impairment of metabolic profile, derive from a review of clinical cases with heterogeneous pathological conditions, such as congenital Hypo-H, Klinefelter syndrome, and male to female or female to male transexualism, associated with a different severity of testosterone deficiency, in a context of either organic or functional hypogonadism, which highlighted that long-term hypogonadism, even if mild, might contribute to the onset of obesity, IR and MetS (51).

A more conspicuous literature exists, concerning the strict and bidirectional relationship between functional Hypo-H, and mainly in LOH, and metabolic disorders, including obesity, MetS, and T2-DM.

Several studies highlighted that obesity is frequently associated with testosterone levels within the hypogonadal range, and a biochemical picture of Hypo-H (41, 52). Indeed, in obese men, SHBG levels may be low, and low TT and/or FT levels have been associated with low or inappropriately normal gonadotropins levels; therefore, a condition defined as male obesity-related Hypo-H might be diagnosed in obese patients presenting with this impairment of HPT axis, along with signs and symptoms of hypogonadism, in absence of an organic impairment of the axis, and recognized causes of hypogonadism (41). Studies addressing TT, FT, and SHBG levels in obese men demonstrated that all were significantly reduced, compared to non-obese men (53, 54); moreover, although SHBG levels were similarly decreased in moderately and severely obese men, TT and FT levels were significantly lower in the latter group, and testosterone deficiency, according to FT levels, was detected only in severely obese subjects (53). Few studies evaluated body fat distribution in relation to androgenic status. Male obesity-related Hypo-H itself might worsen obesity, by promoting fat mass increase, which may in turn significantly worsen the hypogonadal condition; in particular, testosterone deficiency has been demonstrated to increase VAT (55). A cross-sectional cohort study on 217 healthy men highlighted that low TT and SHBG levels were reliable predictors of more severe obesity and greater VAT, therefore also suggesting that age-related differences in testosterone levels could be at least partly determined by the concomitant variation in adiposity (56). Consistently, a different cross-sectional cohort study of 23 healthy men reported that VAT was negatively correlated with TT, FT, and SHBG levels (57). Nevertheless, a case-control study on 26 hypogonadal men failed to detect any difference in VAT, although hypogonadal men displayed increased subcutaneous fat mass (58). An important role in male obesity-related Hypo-H has been attributed to hyperinsulinism, typically associated with obesity, particularly visceral obesity, as demonstrated by a cross-sectional cohort study on 178 men from a larger population-based study on diabetes and cardiovascular disease, in which TT and FT, but not SHBG, levels were negatively correlated with insulin levels, after adjustment for age, obesity, and body fat distribution (59). A different cross-sectional cohort study on 55 obese men confirmed the negative correlation between TT and FT and insulin levels, and reported a negative correlation with insulin levels also for SHBG; these correlations were maintained, independently from BMI (60). Moreover, a cross-sectional cohort study on 1,292 healthy non-diabetic men demonstrated a negative correlation between TT and insulin levels, maintained after adjustment for age and obesity (61). These evidences strongly support the existence of a strict link between testosterone levels and insulin sensitivity, not necessarily correlated with underlying obesity.

Some studies demonstrated that MetS is associated with testosterone deficiency, in particular in presence of obesity (59, 62, 63). Indeed one study on 864 men with mean age of 52 years demonstrated that obese men with MetS, particularly those with severe obesity, have significantly decreased TT levels, compared to healthy men (62); specifically, in multiple linear regression models a significant negative association was found between TT levels and 3 out of 5 of MetS definition criteria, namely, diabetes or fasting glucose levels >110 mg/dl, triglycerides levels ≥150 mg/dl, as well as BMI ≥30 kg/m^2^ (62). Moreover, a population based, prospective cohort study on 1,896 men with mean age of 52 years demonstrated that TT, FT, and SHBG levels were significantly lower in men with MetS compared to men without MetS, and that men in the lowest tertile for FT levels were more likely to have MetS, after adjusting for age and BMI (16). In this study, low testosterone and SHBG levels were associated with MetS independently from BMI, and in particular TT, FT, and SHBG levels were negatively correlated with insulin, glucose, and triglycerides, and positively correlated with HDL-cholesterol levels (16). Lastly, in a different study on 87 healthy men TT, FT, and SHBG levels were found to be positively correlated to total whole body glucose disposal (64).

Several studies reported that T2-DM is associated with testosterone deficiency (2, 65) and that both TT and FT levels, as well as SHBG levels, were reduced in patients with T2-DM (19, 66); moreover, in patients with T2-DM, testosterone deficiency, often associated with low or inappropriately normal levels of gonadotropins, particularly LH, might contribute to worsen sexual dysfunction (67, 68). The first study focusing on Hypo-H in T2-DM, a cross-sectional cohort study performed on 103 men, reported a high prevalence of subnormal FT (33%), and low levels of gonadotropins, confirming the presence of an underlying hypothalamic-pituitary disorder, rather than a defect within the testis (19). Moreover, an inverse relationship was found between TT, FT, and SHBG with BMI (19), also confirmed by subsequent studies (21, 22). A cross-sectional cohort study on 355 men with T2-DM confirmed a high prevalence of Hypo-H in T2-DM patients, defined as low TT and FT levels, and low or inappropriately normal LH levels; moreover, by including the assessment of prevalence of symptoms related to hypogonadism, a prevalence of 17% and 14% of hypogonadism was found, when considering low TT or low FT, respectively (28). A large study on 2098 men demonstrated that the prevalence of Hypo-H, with decreased TT, FT, and SHBG, was significantly higher in T2-DM patients, rather than patients without T2-DM (69). A different cross-sectional study on 580 men reported a prevalence of testosterone deficiency of 43% and 57%, by considering TT and calculated FT, respectively, in T2-DM, and of 7% and 20% in type-1 diabetes mellitus (T1-DM) (66). Nevertheless, another study failed to demonstrate testosterone deficiency occurrence in T1-DM, despite a negative correlation between TT and FT, but not SHBG, levels and BMI was reported (20). Lastly, consistently with different studies in non-diabetic normal or overweight men with or without MetS (70, 71), calculated FT levels were negatively correlated with insulin levels, HOMA-IR and VAT, also in a cross-sectional cohort study on men with T2-DM, further supporting the hypothesis that IR reduces circulating testosterone levels, and that reduced circulating testosterone, in turn, contributes to increase IR (66). Consistently, a cross-sectional cohort study on men with T2-DM and erectile dysfunction reported that 86% of men displaying subnormal FT levels had Hypo-H, suggesting that Hypo-H is the most common gonadal dysfunction in diabetic men affected by erectile dysfunction (68).

In conclusion, consistent evidences from clinical and experimental studies support the concept that Hypo-H may be an independent risk factor for metabolic disorders; moreover, specifically in LOH, a vicious circle hypothesis has been repeatedly demonstrated, linking Hypo-H to obesity, IR, MetS, and T2-DM in a bidirectional relationship, in which visceral obesity and IR seem to be the major pathophysiological players; this evidence confirmed that, besides TT, calculated FT has to be considered as a crucial endpoint to be assessed in patients with metabolic disorders, due to the effect of visceral obesity and IR on SHBG and, consequently, on FT and downstream FT actions.

## Interventional Studies in Patients With Hypogonadism: Effects on Metabolic Disorders

Several studies in cohorts of men with hypogonadism of various etiology, including Hypo-H, Hyper-H, LOH, or mixed cohorts, focused on the effects of TRT on metabolic parameters. Overall, the results of these studies demonstrated that normalization of testosterone levels in obese men reduces body weight, BMI, WC, and fat mass (72–76). Moreover, metanalyses of studies on hypogonadal men with T2-DM highlighted that TRT is associated with marked reduction in WC, as well as indexes of T2-DM, suggesting that TRT may ameliorate visceral obesity and improve metabolic control in patients with hypogonadism (18, 21, 77). In particular, one prospective study on 55 men with mean age 60.5 years and a diagnosis of LOH, generally associated with MetS and T2-DM, demonstrated that a 9-months treatment with long-acting intramuscular testosterone undecanoate (1,000 mg at weeks 0 and 6 and thereafter every 12 weeks) or transdermal testosterone gel 50 mg/day, significantly reduced WC (78). These results were confirmed by a similar single-blind randomized study on 32 hypogonadal men with mean age 56.6 years and MetS and newly diagnosed T2-DM, receiving a combination of 52-weeks transdermal testosterone gel 50 mg/day and supervised diet and exercise or receiving supervised diet and exercise but not testosterone treatment (79). A different, double-blind placebo-controlled study on 184 hypogonadal men with MetS demonstrated a significant improvement in body weight, BMI and WC, following testosterone undecanoate (1,000 mg at weeks 0, 6, and 18) administration, aimed at restoring normal testosterone levels, for 30 weeks (80). Some studies failed to demonstrate a significant decline in fat mass (81–83), or visceral obesity (84–86), despite an overall improvement of body composition following testosterone treatment; the majority of these studies did not address regional fat distribution as an issue. However, several studies demonstrated that testosterone treatment in men with normal and low-normal testosterone levels or clinical hypogonadism improves body composition not only by increasing lean mass, but also by decreasing fat mass. In one double-blind, randomized dose-response study on 54 healthy eugonadal men aged 18–35 years, treatment with weekly injections of testosterone enanthate at different doses (25–600 mg) for 20 weeks significantly increased total body lean mass, as well as appendices and trunk lean mass, at the highest doses (125, 300, 600 mg) (87). On the other hand a double-blind, randomized, placebo-controlled study on 60 non-obese hypogonadal men over 55 years of age demonstrated that transdermal testosterone treatment at 5 mg/day for 52 weeks resulted in a significantly decreased VAT, compared to untreated men, assessed at dual energy x-ray absorptiometry (DEXA), without change in total body or abdominal subcutaneous fat mass, suggesting a protective effect of testosterone specifically from VAT gain (88). Indeed, despite a consistent positive effect of TRT on body composition, few studies addressed site-specific adipose tissue depot after TRT, by reporting controversial results. In particular, one case-control study on 36 hypogonadal men, comprising 29 men with Hypo-H (20 with known cause and 9 idiopathic), and 7 men with Hyper-H, aged 22–68 years (median age 53 years), highlighted that TRT with 100 mg intramuscular testosterone enanthate once per week for 18 months significantly decreased total body and subcutaneous fat mass, whereas VAT was reduced but change did not reach statistical significance, as assessed at quantitative computed tomography (89). Conversely, another double-blind randomized placebo-controlled study on 108 healthy men over 65 years of age, with testosterone levels 1 SD or more below the mean for normal young men (<475 ng/dL), demonstrated that treatment with transdermal testosterone at 6 mg/day aimed at raising testosterone levels to mid-normal young men range, significantly reduced total body fat mass and fat mass within arms and legs depot, but not VAT, as assessed by regional DEXA, during a 36-months treatment (90). By contrast, in two different studies on men over 60 years of age, androgen treatment preferentially induced a reduction in fat mass within the trunk (91), or an equivalent reduction within both the trunk and the appendices (92), as assessed at DEXA and magnetic resonance imaging (MRI). Suggestively, a double-blind randomized placebo-controlled study on 32 hypogonadal and T2-DM-affected men demonstrated that TRT with 250 mg intramuscular testosterone every 2 weeks reversed downregulation of androgen and estrogen receptors, and aromatase expression in the adipose tissue, which characterized this cohort of patients at baseline (93). Inconsistency of results among studies may rely on differences in testosterone formulations, study population, and specific outcome measures of regional fat distribution.

Small interventional studies evaluated the effects of testosterone treatment on insulin sensitivity in different cohorts of patients, including obese, T2-DM, hypogonadal, and eugonadal men; these studies reported conflicting results, probably due to heterogeneity in dose and duration of testosterone treatment, as well as target cohorts. An inverse relationship between insulin sensitivity and testosterone levels was demonstrated by several studies on obese men and men with T2-DM. A controlled trial on 23 middle-aged men with visceral obesity demonstrated that testosterone treatment with 80 mg oral testosterone undecanoate twice a day for 8 months ameliorated insulin sensitivity, as displayed by a significantly increased glucose disposal rate, measured by hyperinsulinemic-euglycemic clamp, and also significantly reduced VAT, without changes in body mass, as assessed at computed tomography (76). A different study on a similar cohort highlighted a more impressive global effect; in particular, testosterone treatment with transdermal testosterone or dihydrotestosterone for 3 months significantly increased glucose uptake, as assessed by hyperinsulinemic-euglycemic clamp and significantly decreased waist/hip ratio, without changes in body fat mass, therefore displaying improvement of insulin sensitivity (73). Additionally, a double-blind, randomized, placebo-controlled trial on 18 healthy non-obese hypogonadal men demonstrated that treatment with dihydrotestosterone gel at 35 mg/day for 3 months, aimed at normalizing testosterone levels, significantly reduced insulin and leptin levels, and improved HOMA-IR (94). A double-blind randomized crossover study on 24 hypogonadal men (2 Hypo-H, 8 Hyper-H, 14 mixed) with T2-DM demonstrated that TRT with 200 mg intramuscular testosterone propionate every 2 weeks for 3 months significantly reduced HbA1c and glucose levels, WC and waist/hip ratio, and significantly improved HOMA-IR, in patients not treated with insulin therapy, with a reduction of insulin dosing in 50% of insulin-treated patients (95). Another double-blind randomized placebo-controlled trial on 84 men with T2-DM (50 eugonadal and 34 with Hypo-H) showed that a 24-weeks TRT with 250 mg intramuscular testosterone cypionate every 2 weeks improved insulin sensitivity, assessed through hyperinsulinemic-euglycemic clamp, and also induced a decrease of subcutaneous fat, measured by DEXA and MRI, although VAT was not affected by TRT (96). Conversely, different studies failed to demonstrate an improvement in insulin sensitivity in different cohorts of patients. In particular, a small uncontrolled study on 30 eugonadal non-obese men aged 27–30 years showed no effect of supra-physiological testosterone treatment with 100 mg or 300 mg intramuscular testosterone enanthate or with 100 mg or 300 mg 19-nortestosterone decanoate administered once per week for 6 weeks on waist/hip ratio, fasting glucose and insulin levels, and response to the oral glucose tolerance test (97). A double-blind randomized placebo-controlled study on 134 non-diabetic non-obese men over or of 60 years of age, with low or low-normal testosterone levels demonstrated that a 36-months testosterone treatment with daily testosterone gel 1%, at initial dose 7.5 g with dose titration according to TT levels, did not improve insulin sensitivity, as demonstrated by unchanged steady-state plasma glucose concentration at equilibrium, a direct measure of the ability of exogenous insulin to mediate disposal of an infused glucose load under steady-state conditions, during which endogenous insulin secretion has been suppressed by octreotide administration (98). A different small uncontrolled study on 10 hypogonadal men with T2-DM failed to find an effect of TRT with 150 mg intramuscular testosterone enanthate every 2 weeks for 6 months, on the control of diabetes, and fasting glucose or insulin levels (99). Lastly, a small uncontrolled study on 10 men aged 19–27 years with idiopathic Hypo-H demonstrated lack of an effect of TRT with intramuscular testosterone enanthate administered at 100 mg dose every 2 weeks (3 injections) and then at 200 mg dose every 2 weeks (3 injections), on waist/hip ratio or IR, measured by hyperinsulinemic-euglycemic clamp (100).

In conclusion, the effect of testosterone on adipose tissue and insulin sensitivity seems to vary according to dose and duration of testosterone treatment, as well as to testosterone type and formulation, and to the characteristics of target populations; it might be hypothesized that the improvement of IR might be determined by an increase in lean body mass, and a decrease in adipose tissue, or, more specifically, by a decrease in VAT, an action which is more remarkable in hypogonadal men.

A summary at a glance of the main interventional studies evaluating the effects of testosterone treatment on anthropometric indexes and metabolic profile, in hypogonadal, and also eugonadal, men, with or without obesity, MetS, and T2-DM, is provided in Table 1.

**Table 1:** Summary at a glance of interventional studies evaluating the effects of testosterone treatment on anthropometric indexes and metabolic profile, in hypogonadal and eugonadal men, with or without obesity, metabolic syndrome, and type-2 diabetes mellitus.

	**Type of study**	**Patients n^°^ and characteristics and intervention groups**	**Age (years)**	**Testosterone treatment (dose, route, duration)**	**Main outcomes**
Saad et al. (78)	P	55 men with LOH, most of which with MetS and T2-DM Group 1: long acting testosterone (*n* = 28) Group 2: testosterone (*n* = 27)	Group 1: m 61 Group 2: m 60	Group 1: testosterone undecanoate 1000 mg at wk 0 and 6, then every 12 wk for 9 mt Group 2: testosterone gel 50 mg/day for 9 mt	BW: Group 1 = ; Group 2 = WC: Group 1 ↓ (*p* < 0.05); Group 2 ↓ (*p* < 0.05); ↓ Group 1 = ↓ Group 2
Heufelder et al. (79)	B Rnd	32 hypogonadal men with MetS and newly diagnosed T2-DM Group 1: diet and exercise plus testosterone (*n* = 16) Group 2: diet and exercise (*n* = 16)	Group 1: m 57 ± 1.4 Group 2: m 56 ± 1.5	Group 1: testosterone gel 50 mg/day for 52 wk	WC: Group 1 ↓; Group 2 ↓; ↓ Group 1 > ↓ Group 2 HI: Group 1 ↓; Group 2 ↓; ↓ Group 1 > ↓ Group 2 (*p* < 0.001) FPI: Group 1 ↓; Group 2 ↓; ↓ Group 1 > ↓ Group 2 (*p* < 0.001) FBG: Group 1 ↓; Group 2 ↓; ↓ Group 1 = ↓ Group 2
Kalinchenko et al. (80)	DB PCT	184 hypogonadal men with MetS Group 1: testosterone (*n* = 113) Group 2: placebo (*n* = 71)	Group 1: m 52 ± 1.6 (R 35–69) Group 2: m 53 ± 2.2 (R 35–69)	Group 1: testosterone undecanoate 1000 mg IM at 0, 6 and 18 wk Group 2: placebo	BW ↓ (*p* < 0.001) WC ↓ (*p* < 0.001) BMI ↓ (*p* < 0.001) WHR ↓ (*p* < 0.05) HI ↓ (*p* < 0.05) FPI= FBG=
Woodhouse et al. (87)	DB Rnd	54 healthy eugonadal men Group 1: testosterone 25 mg (*n* = 11) Group 2: testosterone 50 mg (*n* = 8) Group 3: testosterone 125 mg (*n* = 12) Group 4: testosterone 300 mg (*n* = 10) Group 5: testosterone 600 mg (*n* = 13)	m 27 ± 4 (R 18–35)	Monthly injections of GnRH agonist in all groups Group 1: testosterone enanthate 25 mg IM every wk for 20 wk Group 2: testosterone enanthate 50 mg IM every wk for 20 wk Group 3: testosterone enanthate 125 mg IM every wk for 20 wk Group 4: testosterone enanthate 300 mg IM every wk for 20 wk Group 5: testosterone enanthate 600 mg IM every wk for 20 wk	VAT: Group 1 ↑ (*p* < 0.05); Group 2 ↑ (*p* < 0.05); Group 3 =; Group 4 =; Group 5 = LBM: Group 1 =; Group 2 =; Group 3 ↑ (*p* < 0.01); Group 4 ↑ (*p* < 0.001); Group 5 ↑ (*p* < 0.001)
Allan et al. (88)	DB Rnd PCT	60 non-obese, non-diabetic, hypogonadal men Group 1: testosterone (*n* = 30) Group 2: placebo (*n* = 30)	Group 1: m 62 ± 1.0 Group 2: m 65 ±1.3	Group 1: transdermal testosterone 5 mg/day for 52 wk Group 2: placebo	BW= BMI= WC= FBM= SF= VAT ↓ (*p* = 0.001) LBM ↑ (*p* < 0.01)
Katznelson et al. (89)	CC	Group 1: 36 hypogonadal men (29 Hypo-H; 7 Hyper-H) Group 2: 44 eugonadal men	Group 1: M 53 ± 2 Group 2: M 53 ± 2	Group 1: testosterone enanthate 100 mg IM every wk for 18 mt	FMB ↓ (*p* < 0.01) SF ↓ (*p* < 0.01) VAT=
Snyder et al. (90)	DB RndPCT	108 healthy men with testosterone levels 1 SD or more below the mean for normal young men (<475 ng/dL) (96 men completed the protocol) Group 1: testosterone Group 2: placebo	>65	Group 1: transdermal testosterone 6 mg/day for 36 mt Group 2: placebo	FBM ↓ (*p* = 0.001) SF ↓ (*p* < 0.001) VAT= LBM ↑ (*p* < 0.001)
Ghanim et al. (93)	DB Rnd PCT	64 men with T2-DM Group 1: 32 hypogonadal men (Group 1a: testosterone; Group 1b: placebo) Group 2: 32 eugonadal men	R 30–65	Group 1a: testosterone enanthate 250 mg IM every 2 wk for 22 wk Group 1b: placebo	Group 1 vs. 2: ↓ expression of AR, ERα and aromatase in adipose tissue (*p* < 0.01).Group 1a vs. 1b:↑ expression of AR, ERα and aromatase in adipose tissue (*p* < 0.01)
Simon et al. (94)	DB Rnd PCT	18 non-obese hypogonadal men Group 1: testosterone (*n* = 6) Group 2: DHT (*n* = 6) Group 3: placebo (*n* = 6)	Group 1: m 53 ± 4.2 Group 2: m 52 ± 3.9 Group 3: m 55 ± 3.6	Group 1: testosterone gel 125 mg/day for 3 mt Group 2: DHT gel 35 mg/day for 3 mt Group 3: placebo	Group 2 vs. 3: FPI ↓ (*p* < 0.01) HI ↓ (*p* < 0.01)
Kapoor et al. (95)	DB Rnd Cs	24 hypogonadal men (2 Hypo-H; 8 Hyper-H; 14 mixed) with T2-DM Group 1: testosterone, then placebo (*n* = 12) Group 2: placebo, then testosterone (*n* = 12)	m 64 ± 1.3 (R 52–76)	Testosterone propionate 200 mg IM every 2 wk for 3 mt followed by 1 mt of washout then placebo for 3 mt or vice-versa	WC ↓ (*p* < 0.05) WHR ↓ (*p* = 0.01) HI ↓ (*p* < 0.05) FBG ↓ (*p* < 0.05) HbA1c ↓ (*p* < 0.05)
Dhindsa et al. (96)	DB Rnd PCT	84 men with T2-DM Group 1: 50 eugonadal men Group 2: 34 hypogonadal men (Group 2a: testosterone, *n* = 20; Group 2b: placebo, *n* = 14)	Group 1: m 52 ± 8.9 Group 2: m 55 ± 7.9	Group 2a: testosterone cypionate 250 mg IM every 2 wk for 24 wk Group 2b: placebo	Group 2a vs. 2b: SF ↑ (*p* = 0.01) VAT= LBM ↑ (*p* < 0.01) HI ↓ (*p* < 0.05) FBG ↓ (*p* < 0.05) HbA1c=
Friedl et al. (97)	UNC	30 non-obese eugonadal men Group 1: testosterone 100 mg (*n* = 8) Group 2: testosterone 300 mg (*n* = 7) Group 3: 19-nortestosterone decanoate 100 mg (*n* = 8) Group 4: 19-nortestosterone decanoate 300 mg (*n* = 7)	m 27 ± 5(R 20–37)	Group 1: testosterone enanthate 100 mg IM every wk for 6 wk Group 2: testosterone enanthate 300 mg IM every wk for 6 wk Group 3: 19-nortestosterone decanoate 100 mg IM every wk for 6 wk Group 4: 19-nortestosterone decanoate300 mg IM every wk for 6 wk	WHR= HI= FPI= FBG=
Huang et al. (98)	DB Rnd PCT	134 non-obese, non-diabetic men with low or low-normal testosterone level Group 1: testosterone (*n* = 74) Group 2: placebo (*n* = 60)	Group 1: m 66 ± 5.0 Group 2: m 68 ± 5.1	Group 1: testosterone gel 7.5 g 1% every day for 36 mt Group 2: placebo	IS= FBG=
Corrales et al. (99)	UNC	10 hypogonadal men with T2-DM	m 64 ± 7.9	Testosterone enanthate 150 mg IM every 2 wk for 6 mt	FPI= FBG= HbA1c=
Tripathy et al. (100)	UNC	10 hypogonadal men (Hypo-H)	R19–27	Testosterone enanthate 100 mg IM every 2 wk for 6 wk, then 200 mg IM every 2 wk for 6 wk	WHR= HI=

*CC, case-control; UNC, uncontrolled cohort; P, prospective; PCT, Placebo Controlled Trial; Rnd, randomized; B, blind; DB, double blind; Cs, crossover; Hypo-H, Hypogonadotropic hypogonadism; Hyper-H, Hypergonadotropic Hypogonadism; BW, body weight; WC, waist circumference; BMI, body mass index; WHR, Waist/Hip Ratio; IS, Insulin Sensitivity; HI, HOMA-Index; FBG, Fasting Blood Glucose; HbA1c, Glycated hemoglobin; FPI, Fasting Plasma Insulin; LBM, Lean Body Mass; FBM, Fat Body Mass; SF, Subcutaneous Fat; VAT, Visceral Adipose Tissue; AR, Androgen receptor; ERα, Estrogen Receptor; MetS, Metabolic Syndrome; T2-DM, type-2 diabetes mellitus; R, range; m, mean; M, median; DHT, dihydrotestosterone; IM, intramuscular; wk, week; mt, month; ↓, reduction; ↑, increase; =, no change*.

## Interventional Studies in Patients With Metabolic Disorders: Effects on Testosterone Production

Several studies in cohorts of men with obesity, MetS, T2-DM, or mixed cohorts, focused on the effects of weight loss and/or drugs used in patients with MetS and T2-DM to control metabolic derangements, on testosterone and gonadotropins levels.

Numerous studies assessed the effects of weight loss, obtained through either diet and lifestyle modifications or bariatric surgery, on testosterone and gonadotropins levels, by demonstrating a greater improvement with the latter approach (23, 101). Indeed, a metanalysis of studies on obese men undergoing diet or bariatric surgery concluded that both interventions significantly increased LH, TT, FT, and SHBG levels, with a simultaneous decrease of estradiol levels; in particular, TT variation after low calorie diet-induced or bariatric surgery-induced weight loss reached 2.87 and 8.73 nmol/L, respectively (101). The recognized mechanisms underlying the beneficial effect of weight loss on the androgenic status comprise VAT reduction and consequent decrease of IR, and the reduction of estradiol-induced and leptin-induced negative feedback on the HPT axis (52). Several interventional studies evaluated the effects of drugs used in patients with MetS and T2-DM, namely insulin sensitizers and antidiabetic drugs, on testosterone levels and hypogonadism, and confirmed a strict relationship between obesity and hypogonadism. A small uncontrolled study on 45 men with MetS demonstrated that a 6-months metformin administration at a daily dose starting from 850 mg/day for the first week, gradually increased to 850 mg twice a day in the second week, and subsequently to 850 mg thrice a day, significantly reduced estradiol and SHBG, and increased LH, TT, and FT levels (26). Moreover, in a different small uncontrolled study on 35 men with MetS stratified in 21 eugonadal and 14 hypogonadal men, a 4-months metformin administration at a dose of 850 mg twice daily, associated with normo-caloric diet and physical activity, significantly increased both TT and FT levels, irrespective of gonadal status (102). In one small uncontrolled study on 16 hypogonadal men with T2-DM, a 6-months daily administration of rosiglitazone at the dose of 8 mg significantly increased SHBG, TT and calculated FT levels (103). In a multicenter study on 176 obese men with T2-DM, short-term combined treatment with exenatide plus metformin or glimepiride plus metformin significantly increased TT levels, with a greater efficacy being displayed by the first combination, and testosterone changes being closely correlated to changes in WC (104). These results suggest that antidiabetic drugs with a differential effect on body weight, namely, drugs which increase or reduce body weight, might consistently display a differential effect on androgenic status; nevertheless, scant literature exists investigating the effects of the new classes of antidiabetic drugs with a positive or neutral effect on body weight on testosterone levels. One study on 30 men with obesity-associated hypogonadism demonstrated that liraglutide administered for 16 weeks at a daily dose of 0.6 mg, and weekly titrated up to 3 mg, significantly increased gonadotropins and TT levels (105). Moreover, a different study on 45 obese and diabetic hypogonadal men demonstrated that adding liraglutide to lifestyle changes, metformin and testosterone treatment, in patients with insufficient metabolic control, significantly increased testosterone levels until normalization by means of improved body weight and glycemic control (106).

## Conclusions

The current review highlights that Hypo-H might contribute to the development or worsening of metabolic conditions, mainly through the increase of visceral adiposity and IR, and that metabolic disorders, including obesity, IR, MetS, and T2-DM, contribute to the development or worsening of testosterone deficiency, which is frequently associated with low or inappropriately normal gonadotropins, therefore hinting to Hypo-H, rather than Hyper-H. Moreover, whereas an independent role of testosterone deficiency in the pathogenesis of metabolic disorders has been indirectly suggested in men with Hypo-H, in functional forms of Hypo-H, particularly in LOH, the existence of a bidirectional causal relationship between testosterone and metabolic derangement has been clearly demonstrated. Few interventional studies demonstrate that TRT in hypogonadal men might improve body composition, particularly by decreasing VAT, and might improve insulin sensitivity. On the other hand, correction of metabolic disorders, especially interventions aimed at reducing body weight and IR, ultimately result in an improvement of testosterone levels and HPT axis function. Nevertheless, further observational studies on men with Hypo-H, and larger clinical trials with TRT are required, to definitely clarify the independent pathogenic role of testosterone deficiency in metabolic disorders, in the context of Hypo-H, and to better define the potential beneficial effects of TRT in Hypo-H-related metabolic features.

## Author Contributions

RP conceived all aspects of the manuscript, performed literature search, and wrote the manuscript. DM and ER significantly contributed to manuscript drafting and writing. FG and MM significantly contributed to manuscript revision during peer review process. CdA critically revised the first original draft and any other version of the manuscript before and after peer review process, and provided significant content contribution and English language support. AC critically revised and reviewed the manuscript for important intellectual content. All authors contributed to manuscript revision, read, and approved the submitted version.

### Conflict of Interest Statement

The authors declare that the research was conducted in the absence of any commercial or financial relationships that could be construed as a potential conflict of interest.

## Abbreviations

IR: insulin resistance
MetS: metabolic syndrome
T2-DM: type-2 diabetes mellitus
Hyper-H: hypergonadotropic hypogonadism
Hypo-H: hypogonadotropic hypogonadism
GnRH: gonadotropin releasing hormone
LOH: late onset hypogonadism
TRT: testosterone replacement therapy
TT: total testosterone
FT: free testosterone
SHBG: sex hormone-binding globulin
BMI: body mass index
WC: waist circumference
VAT: visceral adipose tissue; TNF-a
IL-6: interleukin-6
IL-1b: interleukin-1b
LH: luteinizing hormone
HPT: hypothalamus-pituitary-testis
ADT: androgen deprivation therapy
GnRHa: gonadotropin releasing hormone analogs
HDL: high-density lipoprotein
T1-DM: type-1 diabetes mellitus
DEXA: dual-energy x-ray absorptiometry
MRI: magnetic resonance imaging.
